# Functional and Genetic Analyses Unveil the Implication of *CDC27* in Hemifacial Microsomia

**DOI:** 10.3390/ijms25094707

**Published:** 2024-04-26

**Authors:** Wenjie Song, Xin Xia, Yue Fan, Bo Zhang, Xiaowei Chen

**Affiliations:** 1 Department of Otolaryngology-Head and Neck Surgery, Peking Union Medical College Hospital, Peking Union Medical College and Chinese Academy of Medical Sciences, Beijing 100730, China; 2 Key Laboratory of Cell Proliferation and Differentiation of the Ministry of Education, College of Life Sciences, Peking University, Beijing 100871, China

**Keywords:** hemifacial microsomia, *CDC27*, zebrafish, neural crest cell, CRISPR/Cas9, rescue experiments

## Abstract

Hemifacial microsomia (HFM) is a rare congenital genetic syndrome primarily affecting the first and second pharyngeal arches, leading to defects in the mandible, external ear, and middle ear. The pathogenic genes remain largely unidentified. Whole-exome sequencing (WES) was conducted on 12 HFM probands and their unaffected biological parents. Predictive structural analysis of the target gene was conducted using PSIPRED (v3.3) and SWISS-MODEL, while STRING facilitated protein-to-protein interaction predictions. CRISPR/Cas9 was applied for gene knockout in zebrafish. In situ hybridization (ISH) was employed to examine the spatiotemporal expression of the target gene and neural crest cell (NCC) markers. Immunofluorescence with PH3 and TUNEL assays were used to assess cell proliferation and apoptosis. RNA sequencing was performed on mutant and control embryos, with rescue experiments involving target mRNA injections and specific gene knockouts. *CDC27* was identified as a novel candidate gene for HFM, with four nonsynonymous de novo variants detected in three unrelated probands. Structural predictions indicated significant alterations in the secondary and tertiary structures of *CDC27*. *cdc27* knockout in zebrafish resulted in craniofacial malformation, spine deformity, and cardiac edema, mirroring typical HFM phenotypes. Abnormalities in somatic cell apoptosis, reduced NCC proliferation in pharyngeal arches, and chondrocyte differentiation issues were observed in *cdc27^−/−^* mutants. *cdc27* mRNA injections and *cdkn1a* or *tp53* knockout significantly rescued pharyngeal arch cartilage dysplasia, while *sox9a* mRNA administration partially restored the defective phenotypes. Our findings suggest a functional link between *CDC27* and HFM, primarily through the inhibition of *CNCC* proliferation and disruption of pharyngeal chondrocyte differentiation.

## 1. Introduction

Hemifacial microsomia (HFM), the second most common craniofacial birth defect, has an estimated incidence ranging from 1 in 3500 to 1 in 45,000 live births [1]. Also known as craniofacial microsomia, otomandibular dysostosis, or Goldenhar syndrome, HFM is a rare congenital genetic syndrome with developmental abnormalities in the first and second pharyngeal arches. These abnormalities lead to defects in various facial structures, including the external and middle ear, maxilla–zygoma complex, facial nerves, orbits, eyelids, mandible, and masticatory and facial muscles. HFM is part of the oculo-auriculo-vertebral spectrum (OAVS), with additional clinical manifestations in patients often including urogenital anomalies, brain anomalies, microcephaly, heart defects, and short stature [2].

The etiology of HFM, a multifaceted genetic syndrome, involves environmental factors, maternal intrinsic factors, and genetic factors [3]. While most cases are sporadic, reports exist of HFM patients with a family history, suggesting genetic mutations as contributing factors [4,5,6]. A recent study, including 138 sporadic and eight familial craniofacial microsomia patients, established haploinsufficient variants in *SF3B2* as the most prevalent genetic cause of CFM, explaining ~3% of sporadic and ~25% of familial cases [6]. Consequently, variants in various genes or chromosomes, along with genomic imbalances, are posited to be major causal factors of HFM, with environmental factors potentially augmenting the genetic effects. To date, three interconnected pathogenic hypotheses have emerged: (1) vascular abnormalities and hemorrhage, potentially impeding the development of the first and second pharyngeal arches; (2) damage to Meckel’s cartilage, leading to maxillofacial malformations; and (3) disruptions in cranial neural crest cells (CNCCs) development, interfering with maxillofacial bone formation [3].

CNCCs, originating from the ectoderm, comprise a mesenchymal core situated between the ectoderm and endoderm, along with mesoderm cells. They play a crucial role in the development of cartilage bones, the peripheral nervous system, and connective tissues in the head and neck of vertebrates [7,8]. Following gastrulation, CNCCs undergo an epithelial–mesenchymal transition, separating from the neural plate and migrating to the pharyngeal arches. Their development in this region is regulated by both intrinsic patterning information and endodermal signals, including transforming growth factor beta (TGF-β), retinoic acid (RA), fibroblast growth factor (FGF), and WNT [9]. In humans, the first pharyngeal arch gives rise to structures including the zygomatic arch, maxilla, mandible, malleus, and incus, while the second pharyngeal arch forms the stapes, styloid process, and lesser horn of the hyoid bone [9]. This patterning is similarly observed in zebrafish, with the first pharyngeal arch forming the jaw and the second pharyngeal arch producing structures that support the jaw [7].

The present study conducted whole-exome sequencing (WES) on 12 unrelated probands with HFM and their parents (trios), utilizing multiple bioinformatics methods to identify potential pathogenic genetic factors. Additionally, functional studies in zebrafish were carried out to confirm the involvement of these candidate genes in craniofacial cartilage development.

## 2. Results

### 2.1. Demographics and Clinical Characteristics

**Figure 1:**
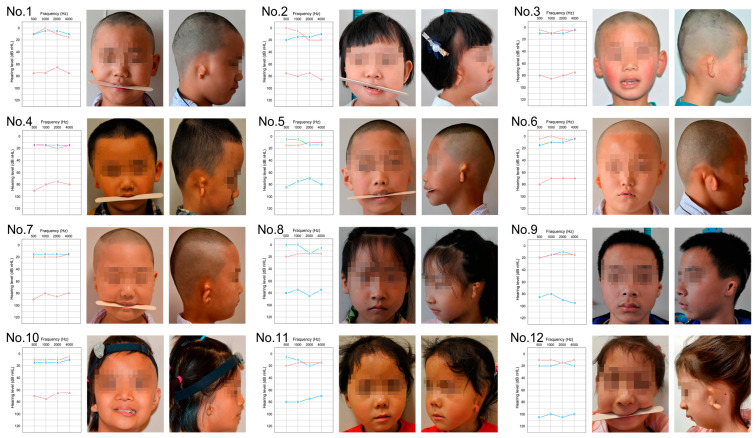
Phenotypic presentation and audiometry findings in 12 probands with hemifacial microsomia (HFM). The characteristic unilateral microtia (classified as Marx grading grade III) and the asymmetrical development of the mandible were presented in all probands, coupled with severe conductive hearing loss on the same side as assessed by pure tone audiometry. The blue line denotes the hearing threshold of the left ear, while the red line represents that of the right ear.

The study enrolled 12 probands (7 males and 5 females) with HFM discordance, along with their unaffected biological parents. The 12 probands were aged between 5 and 8 years, with 5 presenting left-sided malformations and 7 presenting right-sided malformations. The major clinical phenotypes in the probands included gross facial asymmetry, mandibular hypoplasia, unilateral microtia (Marx grading, grade III), and atresia of the ear canal [10]. Pure-tone audiometry tests indicated unilateral moderate to severe conductive hearing loss in all 12 probands (mean air hearing threshold 80 ± 5 dBHL) (Figure 1). No deformities in other systems were detected in the probands, and all parents reported no exposure to known environmental risk factors during pregnancy.

### 2.2. Novel Variant Identification and Molecular Analysis of CDC27

Following comprehensive filtration through databases Kaviar_AF, ExAC, Esp6500, and 1000 Genomes, and an analysis focused on protein-altering variants, a total of 21 nonsynonymous de novo variants from 20 genes were identified (Table 1). Recurrence analysis across various families revealed seven genes (*CDC27*, *COPS7A*, *CTBP2*, *CTDSP2*, *GPM6A*, *RBL1*, and *SNRNP35*) with eight heterozygous mutations, emphasizing the shared genetic features across probands from at least two different families. Among these genes, *CDC27* was notably present in Proband I, III, and VII, displaying three nonsynonymous SNVs and one stopgain variant (Table 2). In Proband I, a nonsynonymous SNV (c.T1424G:p.M475R) in *CDC27* caused an amino acid change from methionine to arginine. In Proband III, two nonsynonymous SNVs (c.T77C:p.F26S and c.T80C:p.L27P) resulted in amino acid changes from leucine to proline and phenylalanine to serine. Proband VII exhibited a stopgain variant (c.T893G:p.L298*). Sanger sequencing verified the absence of these variants in all probands’ parents.

**Figure 2:**
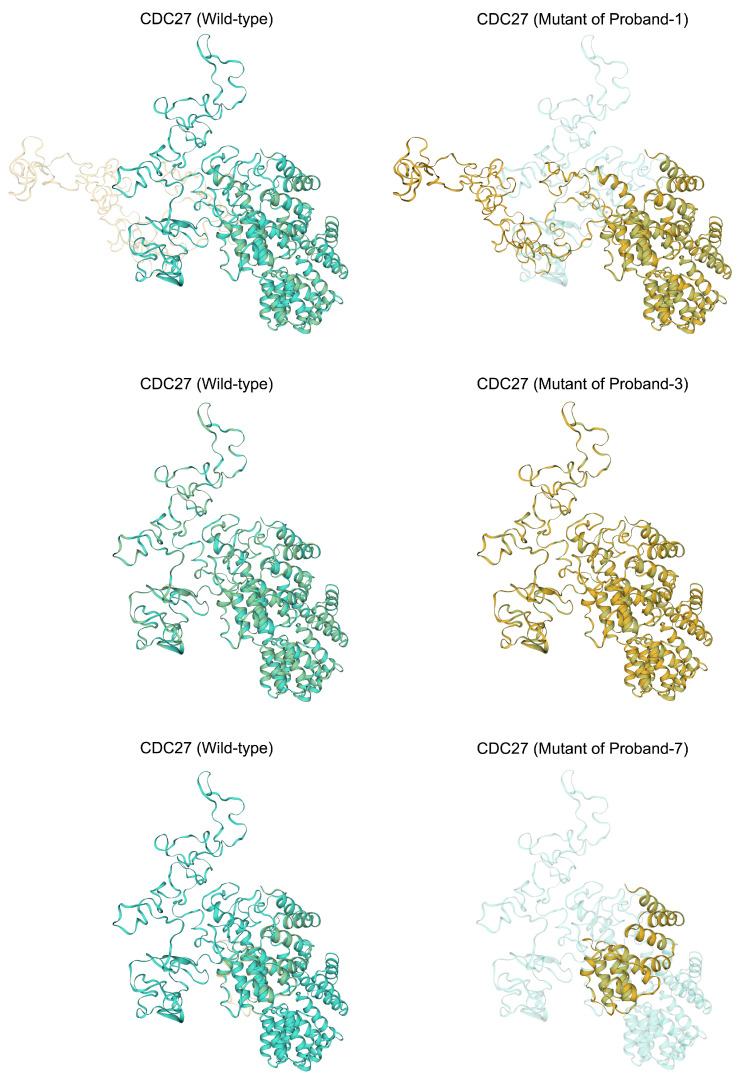
Comparative analysis of predicted tertiary structures in wild-type and mutant protein sequences near mutation sites. This figure presents structural superposition analyses to illustrate the alterations in protein structure resulting from the variants found in Proband I, Proband III, and Proband VII. Significant changes in the tertiary structure of the proteins due to the mutations were found in Proband I and Proband VII. In contrast, the predicted tertiary structure of the protein sequences flanking the variants from Proband III showed no discernible differences when compared to the wild-type structure, indicating that these particular mutations may not significantly alter the protein’s tertiary conformation.

The human *CDC27* gene encodes a protein comprising two conserved domains: the anaphase-promoting complex, cyclosome, subunit 3 (ANAPC3), and the Tetratricopeptide (TPR) repeat. Predictive analyses were conducted on the secondary and tertiary structures of the regions surrounding the identified variants. In Proband I, the de novo variant in the proband introduced an amino acid change from methionine to arginine in the loop area of the TPR repeat domain of the protein. This alteration was predicted to affect downstream secondary structures, potentially disrupting a helix and introducing a strand (Figure S1, upper). The substitution of methionine (a hydrophobic amino acid) with arginine (a basic amino acid) was predicted to alter the local tertiary structure, potentially impacting the conformation of CDC27 (Figure 2, upper). In Proband III, the variants led to amino acid changes from leucine to proline and phenylalanine to serine. These alterations, occurring in the ANAPC3 domain, were predicted to affect upstream secondary structures, potentially disrupting a helix and introducing a coil (Figure S1, middle). However, predictions indicated that the tertiary structure of the sequences surrounding these variants remained similar to the wild type (Figure 2, middle). In Proband VII, the stopgain variant led to premature transcription termination compared to the wild type (Figure S1, lower), resulting in a notable alteration in the predicted tertiary structure (Figure 2, lower).

Protein–protein interaction predictions for CDC27 were conducted with the online STRING software (version 12.0). The interaction network revealed potential direct interactions between CDC27 and proteins like RBX1, PPP2CA, SKP1, and EP300, integral to the TGF-β pathway (Figure S2A). The TGF-β signaling pathway is crucial in regulating the fate of NCCs, as well as osteoblast differentiation and bone formation [11,12]. Collectively, these findings indicate that *CDC27* is a viable candidate gene potentially involved in HFM pathogenesis.

### 2.3. Specifical Expression of cdc27 in the Pharyngeal Arches of Zebrafish

**Figure 3:**
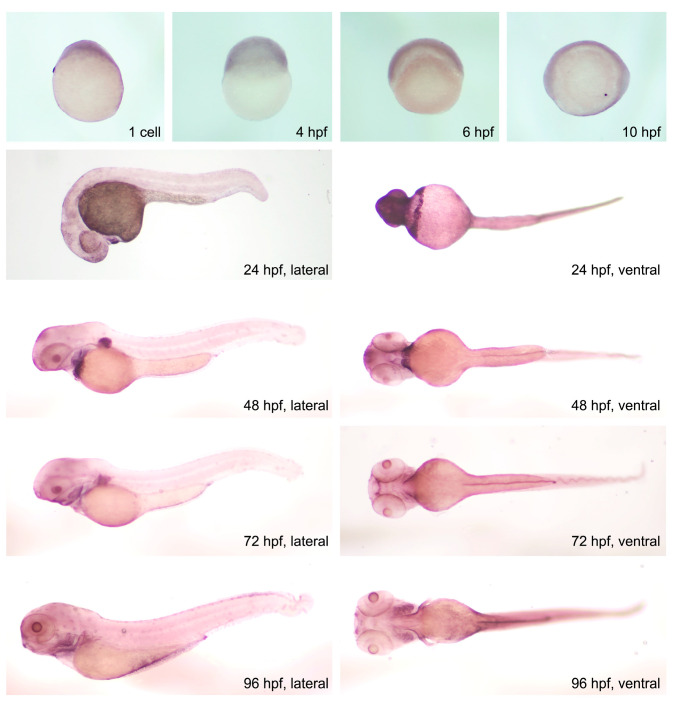
Temporal and spatial expression patterns of *cdc27* in developing zebrafish embryos. In situ hybridization (ISH) results depict the dynamic expression pattern of *cdc27* from the 1-cell stage through to 4 days post-fertilization (dpf), with images presented in both lateral and ventral views. Initially, at the 1-cell stage extending to 10 h post-fertilization (hpf), *cdc27* is broadly expressed throughout the entire embryo. By 1 dpf, there is a notable upsurge in *cdc27* expression, particularly in the head, eyes, trunk, and heart regions. As development progresses to 2 dpf, *cdc27* expression becomes significantly concentrated in the pharyngeal arch region. This focused expression pattern in the pharyngeal arch region becomes even more pronounced from 3 to 4 dpf, underscoring the potential importance of *cdc27* in the development of these specific anatomical structures.

In zebrafish, a homologous gene to human *CDC27*, possessing conserved domains akin to its human counterpart, was identified (Figure S2B). The spatiotemporal expression pattern of cdc27 mRNA during embryonic development in zebrafish was assessed using ISH (Figure 3). The results demonstrated a continuous and ubiquitous expression of *cdc27* mRNA in early zebrafish embryos, spanning from the 1-cell stage to 10 hpf. By 1 dpf, *cdc27* mRNA exhibited widespread expression in the head, eyes, trunk, and heart regions. By 2 dpf, expression became distinctly concentrated in the mandibular region, and between 2 to 4 dpf, the *cdc27* signal intensified specifically within the mandibular region. These findings suggest a pronounced specificity of zebrafish *cdc27* mRNA expression in the mandible, correlating with pharyngeal arch development.

### 2.4. cdc27 Knockout Induces Developmental Malformation and Follow Recessive Inheritance Pattern

**Figure 4:**
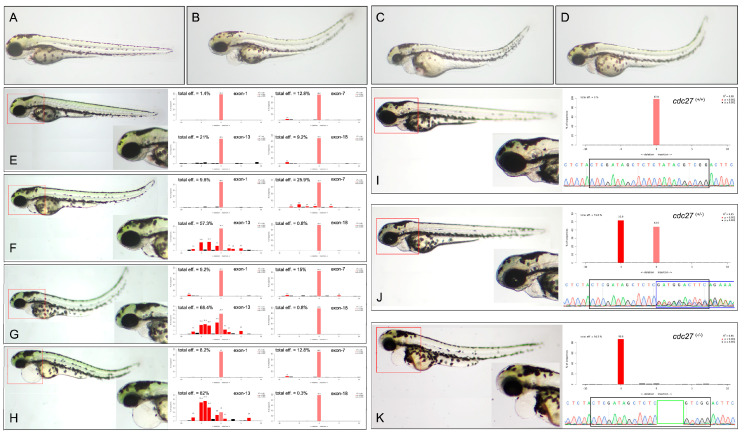
Comparison of phenotypes in wild-type and *cdc27* gRNA injected zebrafish embryos. This figure presents the phenotypic differences observed in wild-type embryos (**A**) and those injected with *cdc27* gRNAs (**B**–**D**) at 3 days post-fertilization (dpf). In over 90% of the gRNA injected embryos, a notable reduction in the overall size of craniofacial structures, along with spine malformation and cardiac edema, was evident compared to control siblings. Panels (**E**–**H**) illustrate the phenotypes and corresponding gene editing efficiency of embryos injected with four different gRNAs at 3 dpf. The severity of mandibular deformity in these embryos exhibited a progressive increase, with gRNA targeting exon-13 showing the highest gene editing efficiency. Moreover, a positive correlation was observed between the editing efficiency of gRNA (exon-13) and the severity of mandibular deformity. Panels (**I**–**K**) depict phenotypes, Sanger sequencing chromatograms, and genotypes of F2 embryos. The F2 generation exhibited two distinct phenotypic categories and three different genotypes: embryos without deformities had either wild-type or heterozygous (5 bp deletion) genotypes, while those with deformities were exclusively homozygous for the 5 bp deletion. The red box highlights enlarged zebrafish structures, the black box denotes the sequence of gRNA (exon-13) (GTC GAT AGC TCT CTA TAC GTC GG), the blue box represents Sanger sequencing bimodal patterns, and the green box indicates the 5 bp deletion sequence (TATAC).

To explore the critical role of *cdc27* in pharyngeal arch development, the Cas9 protein and four different gRNAs, each predicted to be efficacious and without off-target effects, were utilized. These gRNAs were strategically targeted to exon-1, exon-7, exon-13, and exon-18. After specific and quantitative injection into one-cell stage embryos, a significant number of the injected embryos displayed notable reductions in craniofacial structure size, spinal malformations, and cardiac edema at 3 dpf, compared to control siblings (Figure 4A–D). Observations indicated that the gRNA targeting exon-13 had the highest gene editing efficiency, as confirmed by Sanger sequencing and TIDE analysis for genome editing quantification (https://tide.nki.nl/ (accessed on 1 July 2021)) (Figure 4E–H).

To evaluate germline transmission, adult F0 zebrafish were crossbred with TU zebrafish. In F1 embryos, no discernible phenotypes, including pharyngeal arch malformations, cardiac edema, or spinal deformities, were observed. Mutant F1 fish were raised to maturity for subsequent mating to produce F2 offspring, and fin clipping was performed for sequencing purposes. F1 mutant adults were crossbred to yield F2 embryos. In this generation, two phenotypes were observed: Phenotype 1, consistent with wild-type embryos, and Phenotype 2, similar to gRNA-injected embryos, with a phenotype ratio of approximately 3:1. Genotype analysis showed that Phenotype 1 embryos were either wild-type or 5 bp deletion heterozygotes, while Phenotype 2 embryos were exclusively 5 bp deletion homozygotes (Figure 4I–K). Among F2 embryos, the distribution of wild-type, heterozygous, and homozygous genotypes approximated a 1:2:1 ratio, indicative of a recessive inheritance pattern for the characteristic phenotype.

Additionally, the potential impact of the 5 bp deletion variant on the secondary and tertiary structures of Cdc27 in zebrafish was assessed. Our results showed that the 5 bp deletion led to a frameshift, causing premature termination in amino acid translation (Figure S3, upper). The wild-type Cdc27 consists of 790 amino acids, whereas the mutant form contains only 516, leading to changes in the tertiary structure and potentially a loss of function (Figure S3, lower).

### 2.5. Phenotypic Characteristics of cdc27^−/−^ Embryos

#### 2.5.1. Craniofacial Malformations, Spinal Deformities, and Cardiac Edema in *cdc27^−/−^* Embryos

**Figure 5:**
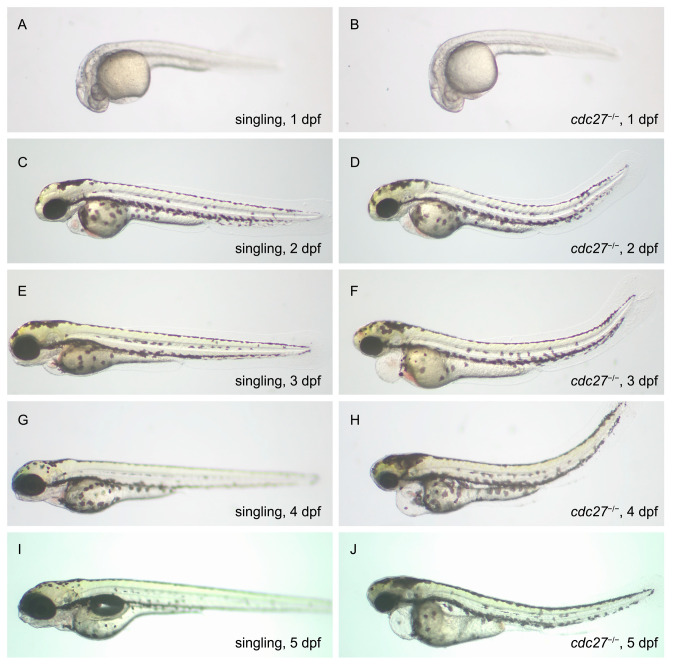
Phenotypic comparison between *cdc27^−/−^* mutants and wild-type zebrafish embryos from 1 to 5 days post-fertilization (dpf). This figure showcases the developmental progression and phenotypic variations observed in *cdc27^−/−^* mutant embryos compared to wild-type embryos from 1 dpf to 5 dpf. At 1 dpf (**A**,**B**), there are no significant phenotypic differences between the two groups. By 2 dpf (**C**,**D**), *cdc27^−/−^* embryos exhibit microcephaly, microphthalmia, and an abnormally curved spine, yet without noticeable abnormalities in the pharyngeal arch. At 3 dpf (**E**,**F**), the mutant embryos begin to show more pronounced phenotypic changes, including the absence of mandible structure formation, exacerbated spinal deformity, and the emergence of cardiac edema. These developmental anomalies, particularly the abnormal mandible development, persist in the mutant embryos at 4 dpf (**G**,**H**) and 5 dpf (**I**,**J**), highlighting the progressive nature of the craniofacial and skeletal deformities in *cdc27^−/−^* mutants.

The phenotypes of *cdc27^−/−^* embryos and their siblings from 1 to 5 dpf are illustrated in Figure 5. At 1 dpf, there were no significant phenotypic differences observed between *cdc27^−/−^* embryos and their siblings. By 2 dpf, mutant embryos began to exhibit microcephaly, microphthalmia, and abnormal spinal curvature, with no pharyngeal arch abnormalities observed in comparison to their siblings. At 3 dpf, while mandible structure formation was observed in the siblings, it was absent in *cdc27^−/−^* embryos. Additionally, spinal deformities in the mutants progressively worsened, and cardiac edema became evident. By 4 and 5 dpf, sibling embryos exhibited gradual mandible maturation, whereas this structure remained abnormal in the mutant embryos. Notably, no significant differences in overall embryo size were observed, suggesting that the mutant phenotypes were not a result of developmental delay.

#### 2.5.2. *cdc27^−/−^* Embryos Feature Pharyngeal Arch Cartilage Dysplasia and Chondrocyte Disorganization

**Figure 6:**
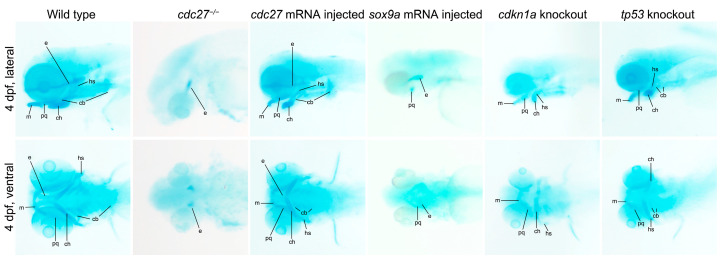
Alcian blue staining of pharyngeal arch cartilages in zebrafish embryos. This figure presents the staining results of pharyngeal arch cartilages using Alcian blue in various groups: wild-type, *cdc27^−/−^*, *cdc27* mRNA injected, *sox9a* mRNA injected, cdkn1a knockout, and *tp53* knockout embryos. Abbreviation: m, Meckel’s cartilages; pq, palatoquadrate cartilages; ch, ceratohyal cartilages; cb, ceratobranchial cartilages; hs, hyosymplectic cartilages; e, ethmoid plate cartilages.

**Figure 7:**
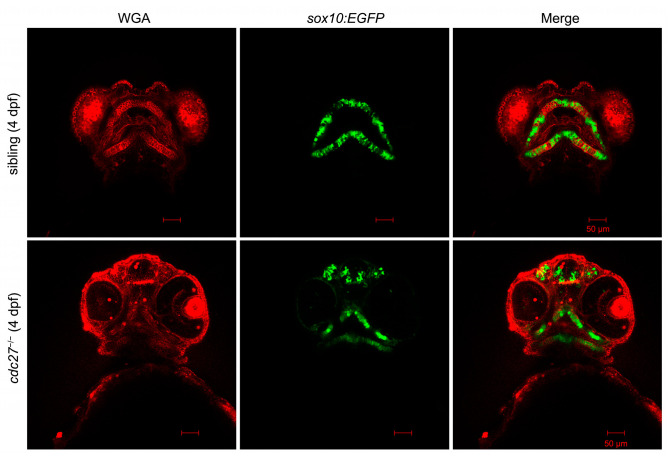
Comparative analysis of chondrocyte morphology in *cdc27* mutants and control siblings using fluorescent WGA staining. This figure illustrates the results of fluorescent wheat germ agglutinin (WGA) staining at 4 days post-fertilization (dpf), showcasing the craniofacial cartilage formation in both control siblings and *cdc27^−/−^* mutant embryos. In the control siblings, the craniofacial cartilage demonstrates a highly consistent and stereotypical shape, akin to a “stack of pennies” where elongated and slender chondrocytes are meticulously arranged in layers, forming the individual cartilage elements. In stark contrast, the *cdc27^−/−^* embryos exhibit significantly altered chondrocyte morphology: the chondrocytes within the cartilage are noticeably smaller and the overall cartilage structures are markedly deformed, deviating substantially from the organized arrangement seen in the control siblings.

Alcian blue staining at 4 dpf showed an absence of specific cartilages including Meckel’s (M), palatoquadrate (pq), ceratohyal (ch), hyosymplectic (hs), and ceratobranchial (cb1-5), along with severely hypoplastic ethmoid plate cartilages (e), in *cdc27^−/−^* embryos compared to wild types (Figure 6, first and second columns). It was hypothesized that altered chondrocyte arrangement in *cdc27^−/−^* embryos might contribute to the observed deformities in pharyngeal cartilage. To further investigate, fluorescent WGA staining was employed to visualize glycoproteins in chondrocyte membranes. At 4 dpf, siblings exhibited a consistent, thin, and elongated chondrocyte structure, forming a “stack of pennies” organization (Figure 7, first line). However, in *cdc27^−/−^* embryos, cartilage elements appeared significantly distorted compared to control siblings (Figure 7, second line). To confirm that the pharyngeal arch cartilage dysplasia was directly caused by the *cdc27* knockout, in vitro synthesized *cdc27* mRNA was injected into one-cell stage *cdc27^−/−^* embryos. Subsequent Alcian blue staining at 4 dpf showed that overexpression of *cdc27* mRNA could effectively rescue the defective pharyngeal arch cartilage phenotypes in *cdc27^−/−^* embryos (Figure 6, third column).

### 2.6. Impact of cdc27 Knockout on NCCs and Pharyngeal Arch Primordia Formation

**Figure 8:**
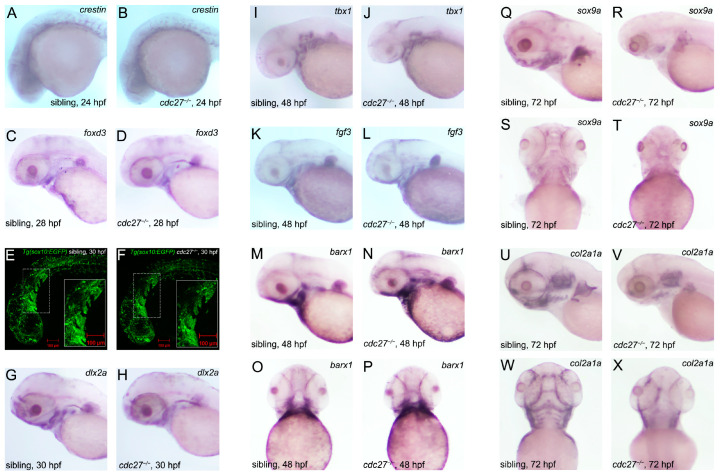
Investigating the impact of *cdc27* knockout on pharyngeal pouches, neural crest cells (NCCs), and pharyngeal cartilage development in zebrafish embryos. This figure presents a series of in situ hybridization (ISH) and fluorescence imaging analyses to assess the developmental changes in *cdc27^−/−^* embryos compared to control siblings. (**A**–**D**) ISH results with crestin and foxd3 probes at 24 h post-fertilization (hpf) and 28 hpf. The staining patterns for *crestin* and *foxd3*, markers for NCCs, show no noticeable differences between mutant embryos and their control siblings. (**E**,**F**) Fluorescence imaging of *sox10*-labeled NCCs in the pharyngeal arch region, marked by a white dotted box and enlarged in the solid white box. Green fluorescence signals indicating NCCs are similar in both *cdc27^−/−^* embryos and siblings. (**G**,**H**) ISH with the *dlx2a* probe at 30 hpf. Expression of *dlx2a*, a gene involved in craniofacial development, appears similar in both mutant and control embryos. (**I**–**L**) ISH with *tbx1* and *fgf3* probes at 48 hpf. The results show no significant differences in the segmentation and number of pharyngeal pouches between *cdc27^−/−^* embryos and siblings. (**M**–**P**) ISH with the barx1 probe at 48 hpf. No significant variation is observed in *barx1* expression between mutant and control embryos. (**Q**–**X**) ISH with *sox9a* and *col2a1a* probes at 72 hpf. While *sox9a* expression is notably reduced in mutants, the expression of *col2a1a*, a marker for cartilage, is absent in the hypopharyngeal arches of the mutant embryos compared to controls, indicating a significant disruption in cartilage development.

In this study, ISH was utilized to monitor the expression of CNCC migration and specification marker genes, including crestin and *foxd3* (expressed in the neural crest from 19 hpf during CNCC migration to the cranial area), and *dlx2a* (expressed in all CNCCs in the pharyngeal arches at 30 hpf post migration completion) [11]. At 24 hpf and 28 hpf, respectively, no significant differences in crestin and foxd3 expression were observed between mutant embryos and their control siblings, indicating that *cdc27* knockout did not impact NCC formation (Figure 8A–D). To observe CNCC migration, the transgenic fish Tg(*sox10*:EGFP), with labeled NCCs, was employed. At 30 hpf, similar green fluorescence signals were observed in the pharyngeal arch region of both mutants and siblings (Figure 8E,F), suggesting that CNCC migration was unaffected by *cdc27* knockout. Furthermore, *dlx2a* expression domains at 30 hpf were found to be comparable between mutant embryos and control siblings, signifying that CNCC specialization remained unaffected by *cdc27* knockout (Figure 8G,H).

### 2.7. Effect of cdc27 Knockout on Pharyngula Formation and Mesenchymal Cell Aggregation

To evaluate the impact of *cdc27* knockout on pharyngula formation, tbx1 and fgf3, specific marker genes, were employed as probes in ISH. The results indicated no significant differences in the segmentation or number of pharyngula between mutant embryos and control siblings, suggesting that *cdc27* knockout did not influence pharyngula formation (Figure 8I–L). Additionally, ISH using barx1 as a probe in 48 hpf embryos revealed no significant differences in barx1 expression between mutants and siblings (Figure 8M,N), indicating that *cdc27* knockout did not affect mesenchymal cell aggregation in the pharyngeal arch primordium.

### 2.8. Impact of cdc27 Knockout on Pharyngeal Arch Cartilage Differentiation

**Figure 9:**
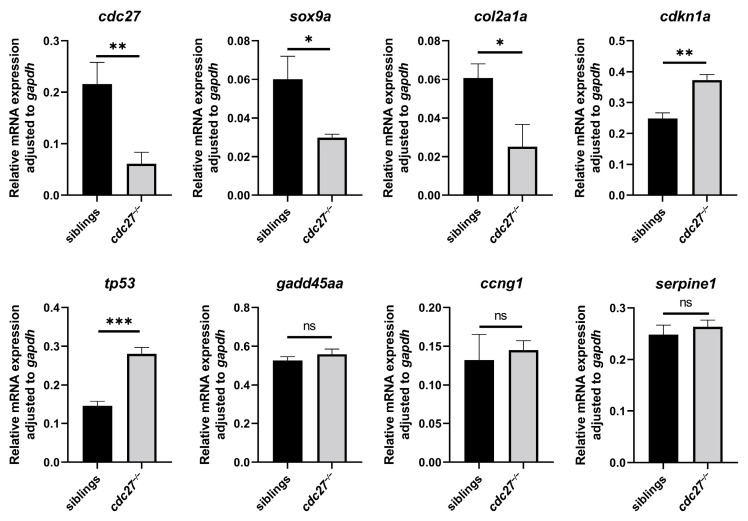
RT-qPCR analysis of gene expression in *cdc27^−/−^* mutant and control sibling zebrafish embryos. This figure illustrates the relative mRNA levels of several key genes, including *cdc27*, *sox9a*, *col2a1a*, *cdkn1a*, *tp53*, *gadd45aa*, *ccng1*, and *serpine1*, as determined by RT-qPCR analysis. In the *cdc27^−/−^* embryos, there is a notable decrease in the mRNA levels of *cdc27*, *sox9a*, and *col2a1a*, suggesting altered gene expression related to craniofacial development and cartilage formation. Conversely, the mRNA levels of cdkn1a and *tp53* are elevated in the mutants, indicating potential compensatory mechanisms or stress responses. Notably, the mRNA levels of *gadd45aa*, *ccng1*, and *serpine1* show no significant differences between the control siblings and *cdc27^−/−^* embryos, implying a selective impact of the *cdc27* mutation on specific gene expressions. Note: *, *p* < 0.05; **, *p* < 0.01; ***, *p* < 0.001; ns, *p* > 0.05.

ISH was utilized to monitor the expression of *sox9a*, a marker of cartilage differentiation, and *col2a1a*, a marker of cartilage. At 72 hpf, *sox9a* expression was observed in similar domains in both mutant and control siblings; however, expression levels in mutants were significantly lower than in control siblings (Figure 8Q–T). In control siblings, *col2a1a* expression at 72 hpf was evident in the hypopharyngeal arches, and structures such as Meckel’s, ceratohyal, and ceratobranchial were clearly distinguishable, indicating the onset of mandible formation. Conversely, col2a1a expression was absent in the hypopharyngeal arches of mutant embryos (Figure 8U–X). Additionally, qRT-PCR analysis revealed lower mRNA levels of *cdc27*, *sox9a*, and *col2a1a* in *cdc27^−/−^* embryos compared to controls (Figure 9, ** p = 0.005, * p = 0.0121, and * p = 0.0105). To ascertain whether reduced sox9a transcription in *cdc27^−/−^* embryos led to pharyngeal arch cartilage deformities, in vitro synthesized *sox9a* mRNA was injected. Alcian blue staining showed that *sox9a* mRNA injection could only partially rescue the defective pharyngeal arch cartilage phenotypes in *cdc27^−/−^* embryos (Figure 6, fourth column). Collectively, these findings imply that *cdc27* knockout may influence cartilage differentiation within the pharyngeal arch.

### 2.9. cdc27 Knockout Leads to a Decrease in the Proliferation of NCCs in the Pharyngeal Arches and an Increase in Somatic Cell Apoptosis

**Figure 10:**
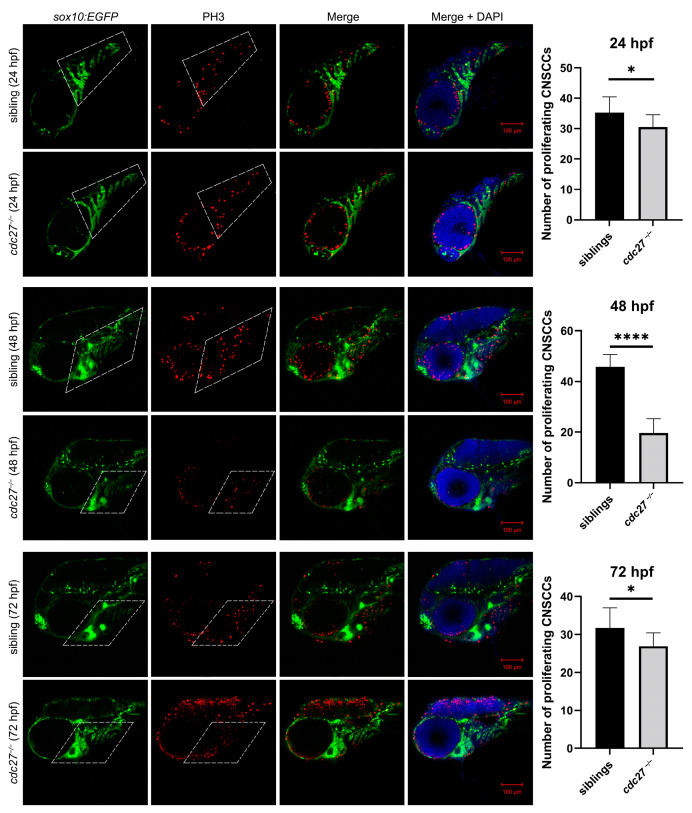
Comparison of CNCC proliferation in *cdc27^−/−^* mutants and control siblings at 24 h post-fertilization (hpf), 48 hpf, and 72 hpf. This figure presents immunofluorescence results depicting cell proliferation through antiphosphohistone H3 (PH3) staining in *cdc27^−/−^* mutants and their control siblings at different developmental stages. The merged images highlight the anti-PH3 signals in the CNCCs located in the pharyngeal arch region, marked by a dotted box. Note: *, *p* < 0.05; ****, *p* < 0.0001.

**Figure 11:**
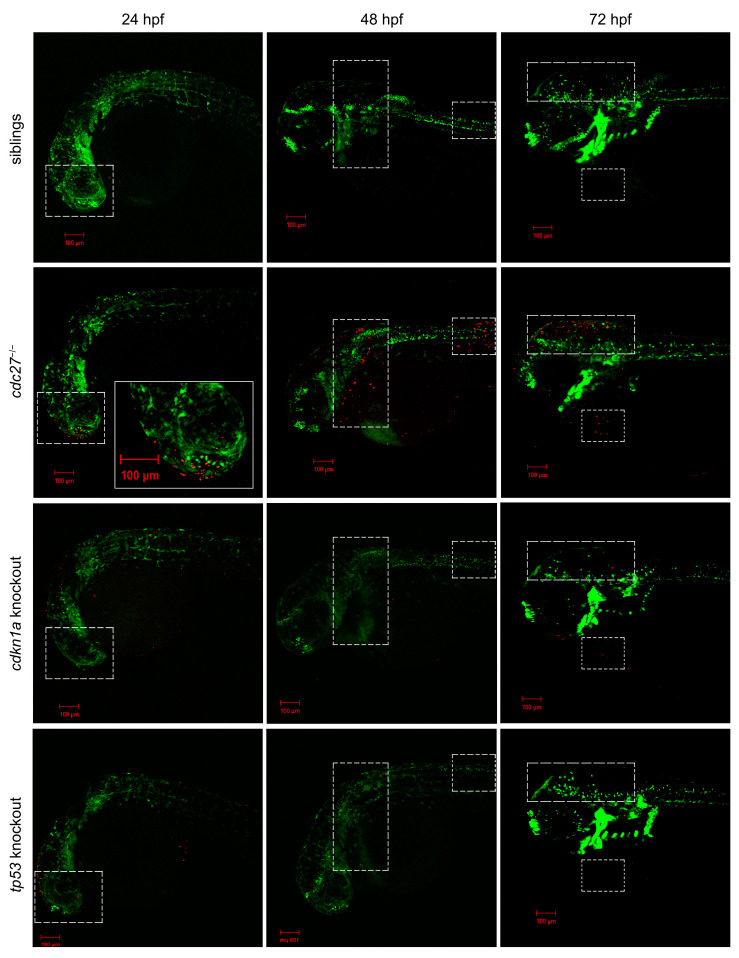
Analysis of somatic cell apoptosis in wild-type, *cdc27^−/−^*, cdkn1a knockout, and tp53 knockout zebrafish embryos at 24 h post-fertilization (hpf), 48 hpf, and 72 hpf. This figure displays the results of TUNEL staining, a method used to detect apoptosis in somatic cells, across different groups of embryos at various developmental stages. In the *cdc27^−/−^* mutant embryos, TUNEL staining signals, indicative of apoptotic cells, are observed in the head, trunk, spine, encephalocoele, and ventricle regions at 24, 48, and 72 hpf, respectively. In contrast, control siblings and embryos with *cdkn1a* or *tp53* knockouts show no apparent apoptotic signals. The dotted boxes highlight areas of TUNEL staining, while the solid boxes provide enlarged views of these regions, emphasizing the specific areas where apoptosis is detected in the mutants.

We hypothesize that the distinct phenotypes observed in the mutant embryos might be attributable to abnormal cell proliferation and/or apoptosis, especially in NCCs. To identify proliferating and apoptotic cells, PH3 immunostaining and TUNEL staining were conducted on whole embryos at 24 hpf, 48 hpf, and 72 hpf. Tg(*sox10*:EGFP) embryos were used to confirm that the observed signals were located in NCCs or other somatic cells. Results showed a significant decrease in NCC proliferation signals in the pharyngeal arch of *cdc27^−/−^* mutants compared to control siblings at 24, 48, and 72 hpf (Figure 10, * p = 0.0326, **** p < 0.0001, * p = 0.0288). Additionally, cell apoptosis was significantly altered in *cdc27^−/−^* mutants. In control siblings, no apparent signs of cell apoptosis were detected; however, *cdc27^−/−^* embryos exhibited apoptosis signals in somatic cells, including in the head, trunk, spine, encephalocoele, and ventricle at 24, 48, and 72 hpf (Figure 11). Significantly, the regions exhibiting apoptotic signals corresponded with the observed abnormal phenotypes, except for in the pharyngeal arch.

### 2.10. RNA-Seq Analysis

#### 2.10.1. Identification of Differentially Expressed Genes

To gain insights into the molecular-level phenotype of the mutant embryo, a comprehensive transcriptome analysis was performed on the entire embryo in both the mutant and control groups. Adapters and low-quality data were removed, resulting in a clean read ratio of over 99% for all groups, ensuring the reliability of the RNA-sequencing data. The clean reads from each group were aligned to the zebrafish reference genome, achieving a mapping rate exceeding 92%. The principal component analysis (PCA) revealed clear differentiation between the control and mutant groups, evidenced by distinct clusters for each (Figure S4A), facilitating the identification of notable differences between the two groups. In total, 334 significantly differentially expressed genes (DEGs) were identified, including 225 upregulated and 109 downregulated DEGs, each exhibiting fold changes greater than 1.0 and *p*-values less than 0.05 (Figure S4B and Table S4).

#### 2.10.2. KEGG Enrichment Analysis for DEGs

We employed the online software KOBAS (version 3.0) to conduct KEGG enrichment analysis on the identified DEGs. The enriched KEGG pathways of DEGs (*p*-value < 0.05) are illustrated in Figure S5. Among the DEGs, several were identified as being involved in the p53 pathway, a pathway previously demonstrated to have a close correlation with zebrafish maxillofacial development. These DEGs include *cdkn1a*, *serpine1*, *gadd45aa*, *tp53*, and *ccng1*, each of which exhibited upregulation in the mutant group. Moreover, we utilized the online STRING software (version 12.0) to predict protein–protein interactions between these five proteins and Cdc27. The resulting interaction network indicates that Cdc27 may directly interact with Cdkn1a and Ccng1, and potentially associate with Gadd45aa, Serpine1, and Tp53 (Figure S6).

#### 2.10.3. Evaluation of DEGs Expression Involved in p53 Pathway

We assessed the expression levels of cdkn1a, ccng1, serpine1, gadd45aa, and tp53, all involved in the p53 pathway, via qRT-PCR analysis. Among these genes, only cdkn1a and tp53 showed significantly higher expression in the *cdc27^−/−^* embryos (Figure 9; ** p = 0.0012 and *** p < 0.0003). Conversely, the expression levels of gadd45aa, ccng1, and serpine1 exhibited no significant differences between the two groups (Figure 9; *p* = 0.1630, *p* = 0.5629 and *p* = 0.3157, respectively).

### 2.11. cdkn1a and tp53 Knockout Reverses Dysplasia of Pharyngeal Arch Cartilage in cdc27^−/−^ Embryos

**Figure 12:**
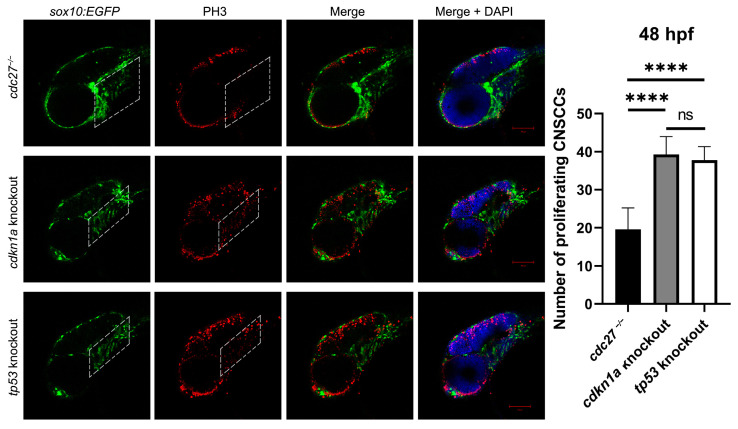
Comparative analysis of CNCC proliferation in *cdc27^−/−^*, *cdkn1a*, and *tp53* knockout embryos at 48 h post-fertilization (hpf). This figure displays the results of immunofluorescence staining using antiphosphohistone H3 (PH3), a marker of cell proliferation, in three groups of zebrafish embryos: *cdc27^−/−^* mutants, *cdkn1a* knockouts, and *tp53* knockouts. The merged images highlight anti-PH3 signals within the CNCCs in the pharyngeal arch region, marked by a dotted box. Notably, the intensity of these signals in cdkn1a and *tp53* knockout embryos is increased compared to the *cdc27^−/−^* embryos, indicating a higher rate of cell proliferation in the pharyngeal arches of these knockouts. This suggests a variation in the proliferation rates of CNCCs due to different genetic modifications, with *cdkn1a* and *tp53* knockouts exhibiting enhanced proliferation relative to the *cdc27^−/−^* mutants. Dotted box: pharyngeal arch reign. Note: ****, *p* < 0.0001.

We achieved the knockout of *cdkn1a* and *tp53* genes by using the Cas9 protein mixed with four gRNAs. Alcian blue staining demonstrated that the knockout of *cdkn1a* and *tp53* genes partially restored the defective phenotypes of pharyngeal arch cartilage in *cdc27^−/−^* embryos (Figure 6, fifth column and sixth column). Furthermore, we performed PH3 immunostaining and TUNEL staining on the entire embryos of cdkn1a-knockout *cdc27^−/−^* Tg (*sox10*:EGFP) and *cdc27^−/−^* Tg (*sox10*:EGFP) embryos at 72 hpf, respectively. Confocal imaging analysis revealed that the proliferation signals of NCCs in the pharyngeal arch of *cdkn1a*-knockout and *tp53*-knockout *cdc27^−/−^* embryos were restored, and the extent of cell apoptosis was markedly reduced in comparison to *cdc27^−/−^* embryos (Figure 12).

In summary, the knockout of *cdc27* reduces the transcription of *sox9a*, thereby hindering the differentiation of pharyngeal arch cartilage. Additionally, *cdc27* knockout leads to overexpression of *cdkn1a* and *tp53*, resulting in the abnormal activation of the p53 signaling pathway, which is characterized by decreased cell proliferation and increased apoptosis. These cellular abnormalities consequently result in malformations in *cdc27^−/−^* embryos.

## 3. Discussion

This study identified nonsynonymous de novo variants in ten genes, shared by at least two affected probands: *CDC27*, *COPS7A*, *CTBP2*, *CTDSP2*, *GPM6A*, *RBL1,* and *SNRNP35*. These variants were detected via WES across 12 trio families. Utilizing bioinformatic analyses, structural predictions, and functional zebrafish studies, it was predicted that the c.T1424G:p.M475R and c.T893G:p.L298* variants in *CDC27* are likely associated with HFM. Despite involving a single base mutation, in silico predictions suggested these variants could induce significant alterations in the secondary and tertiary structure of the CDC27 protein, potentially leading to substantial functional changes. The functional analysis of *CDC27* in zebrafish, which demonstrated typical phenotypic changes after *cdc27* knockout, further corroborated these findings.

The *CDC27* gene consists of 33 specific exons that undergo alternative splicing, resulting in multiple transcripts, including 13 spliced mRNAs assumed to produce functional proteins. The major functional isoforms of *CDC27* comprise 830 and 824 amino acids, encoded by 19 exons, and feature two tetratricopeptide repeat (TPR) domains, with five TPR motifs in the N-terminal and nine in the C-terminal domain. *CDC27* serves as a core component of the Anaphase-Promoting Complex/Cyclosome (APC/C), primarily controlling cell cycle transitions during cellular division [13]. Prior research has indicated that *CDC27* may function as either a tumor suppressor or an oncogene in various neoplasms, and the activation of APC/C is thought to be linked to their pathogenesis [14,15]. The phosphorylation of CDC27 is crucial for APC/C activation, a process facilitated by TGF-β [16]. The TGF-β signaling pathway plays a crucial role in various biological processes, such as cell proliferation, apoptosis, differentiation, migration, and the development of congenital craniofacial malformations [17,18,19]. Bioinformatics analyses have predicted potential crosstalk of CDC27 with four proteins in the TGF-β pathway: RBX1, PPP2CA, SKP1, and EP300. Mutants deficient in TGF-beta-activated kinase 1 (Tak1) display a round skull, hypoplastic maxilla, and mandible [12], indicating the importance of the TGF signaling pathway in craniofacial development. Furthermore, the TGF signaling pathway is essential for osteoblast differentiation and bone formation [11]. As a downstream molecule of the TGF-β pathway, CDC27 activates APC/C (ubiquitin ligase) under the influence of TGF-β through phosphorylation, thus facilitating the ubiquitin–proteasome system. This may impact the cell cycle process, impede protein degradation, and contribute to abnormalities in CNCC proliferation, differentiation, and somatic cell apoptosis.

This study focused on investigating the impact of *cdc27* knockout on cartilaginous elements in mutant embryos. Interestingly, it was observed that the residual cartilaginous structures in these embryos formed appropriate patterns. Additionally, structures derived from both the first and second pharyngeal arches were affected, and craniofacial development markers exhibited proper expression in pertinent areas. These findings imply that although *cdc27* may not be essential for the migration, aggregation, or specification of CNCCs, it appears to play a key role in regulating their quantities. Supporting this hypothesis is the notable difference in the expression of *sox9a*, a marker gene for cartilage differentiation, between wild-type and mutant embryos. Prior research has demonstrated that in *sox9a* zebrafish mutants, neural crest specialization and migration proceed normally. However, these mutants exhibit reduced *col2a1* expression and almost completely lack skull cartilage, with only a few cells of the hyoid bone remaining intact [20]. The findings of this study indicate that *sox9a* is not essential for neural crest specialization or pharyngeal arch cartilage aggregation. Nevertheless, *sox9a* seems to play a regulatory role in the subsequent differentiation of neural crest cells into chondrocytes. To delve deeper into the relationship between *cdc27* and *sox9a*, *sox9a* mRNA was injected into *cdc27^−/−^* embryos at the one-cell stage. This intervention partially ameliorated the mutant phenotype, albeit to a limited extent. It is postulated that the limited efficacy of this rescue could be attributed to the gradual degradation of *sox9a* mRNA in the embryos during development from the one-cell stage to 3 dpf.

In the *cdc27^−/−^* embryos, both craniofacial cartilage and CNCC marker domains, along with PH3 immunofluorescence signals, were diminished, indicating a reduction in CNCC numbers. The observed reduction in CNCC numbers may be attributed to increased apoptosis and reduced proliferation, both known to be associated with various craniofacial anomalies. Previous studies have shown that increased apoptosis of CNCCs can affect secondary palate fusion and is associated with significant mid-facial anomalies, including cleft palate [21,22]. Likewise, a reduction in CNCC proliferation due to a vwa1 variant has been functionally associated with HFM [4]. Moreover, the loss of tln1 has been proposed to lead to a shortened palate and altered Meckel’s cartilage in zebrafish, with tln1 being crucial for CNCC proliferation during palate morphogenesis [23]. In this study, a noticeable decrease in CNCC proliferation was observed in mutant embryos, which could be a critical factor contributing to the developmental abnormalities observed in the mutant maxillofacial region.

By conducting RNA sequencing analysis, we discovered a notable enrichment of genes highly expressed in *cdc27^−/−^* embryos within the p53 signaling pathway, including *cdkn1a* and *tp53*, when compared to their wild-type siblings. It revealed that the proliferation signals of NCCs in the pharyngeal arch were restored after *cdkn1a* and *tp53* were knockout in *cdc27^−/−^* embryos, and the extent of cell apoptosis was markedly reduced when compared to *cdc27^−/−^* embryos. These findings were consistent with previous studies. A prior study established that improper activation of p53 pathway is linked to various human congenital diseases, including craniofacial deformities [24]. It was proved that *CDC27* expression regulates *CDKN1A*, a cyclin-dependent kinase inhibitor in the cell cycle, within the p53 signaling pathway. This, in turn, regulates ID1 expression, a factor controlling the G1/S transition in the cell cycle, thereby influencing the G1 to S phase transition [25,26]. Craniofacial defects are a common manifestation in Treacher Collins syndrome (TCS), primarily caused by heterozygous mutations in *TCOF1* [27]. *Tcofl1*^+/−^ mouse embryos show increased *p53* immunostaining and heightened apoptosis in neural epithelial cells, leading to a marked reduction in NCC numbers. This reduction in NCCs is likely a direct result of the augmented apoptosis in neural epithelial cells. Significantly, reducing *p53* expression can ameliorate neural epithelial cell apoptosis, diminished neural crest formation, and craniofacial defects in *Tcofl1*^+/−^ mice [28]. In TCS, some patients possess *POLR1C* and *POLR1D* mutations. Knocking down or knocking out *p53* can rescue neuronal epithelial cell apoptosis and craniofacial defects in zebrafish with *polr1c* or *polr1d* mutations [29,30].

While no direct disease-causing variants of *CDC27* for HFM or other congenital craniofacial malformations have been conclusively identified, this study demonstrates that *cdc27* knockout in zebrafish results in phenotypic changes like craniofacial malformations, spinal deformities, and cardiac edema, aligning with typical HFM phenotypes. Additionally, the identification of nonsynonymous de novo variants in three unrelated sporadic cases lends support to the hypothesis that *CDC27* could be a pathogenic gene for HFM, especially considering the condition’s low incidence. Further exploration is required to elucidate the mechanism of *CDC27* mutation in HFM. Meanwhile, disease condition was observed in patients with *CDC27* heterozygous variant. In contrast, in zebrafish, heterozygotes showed no pathological phenotype, whereas homozygous mutation well recapitulated the disease phenotype. The observed haplosufficiency in zebrafish, in contrast to humans, deserves further future study.

The current study has several limitations. Firstly, it relies on a relatively small cohort of HFM cases. Although the discovery of *CDC27* variants in these cases is compelling, a larger sample size would strengthen the evidence supporting the involvement of *CDC27* in HFM and facilitate the identification of other potentially implicated genes. Additionally, while the zebrafish model provides valuable insights, the lack of functional validation in mammalian models, particularly those closely resembling human facial development, is a limitation. Lastly, although the study underscores the potential clinical implications of *CDC27* in HFM, it does not explore therapeutic interventions. Further research is necessary to translate these findings into actionable treatments or preventive measures for HFM.

## 4. Materials and Methods

### 4.1. Study Participants

This study adhered to the principles outlined in the Declaration of Helsinki. Written informed consent was obtained from all participants, and the study protocol received approval from the Ethics Committee of Peking Union Medical College Hospital (No. JS796). Furthermore, guardians granted permission to disclose identifiable individuals in photographs and to collect blood samples for genetic analyses. Patients displaying the typical phenotype of HFM, characterized by unilateral microtia (Marx grading, Grade III) and ipsilateral hemifacial hypoplasia, along with their unaffected biological parents, were recruited for the study. All HFM patients had been hospitalized at the Department of Otolaryngology, Peking Union Medical College Hospital, between January 2017 and December 2019. Family histories were obtained, and clinical features of all participants were evaluated. Patients diagnosed with syndromes other than HFM or those with a positive family history were excluded. Finally, the included probands and their biological parents underwent trio-whole-exome sequencing (trio-WES).

### 4.2. Whole-Exome Sequencing

Peripheral blood samples were collected from the recruited probands and their biological parents, from which total genomic DNA was extracted. Sufficient quality genomic DNA samples underwent random fragmentation using the TIANamp Blood DNA Kit (Tiangen, Beijing, China). Library construction was performed on the resulting double-stranded DNA fragments, targeting a size range of 200 to 300 bp. After the end repair and A-tailing steps were completed, sequencing adaptors were added to both ends of the fragments. To tag the DNA of all subjects, the adaptor-bonded products were amplified using index-tagged primers. The amplified products were then purified using the QIAquick PCR Purification kit (QIAGEN, Frankfurt, Germany). Genomic DNA enrichment and sequencing involved sonication (cat: FB705, Thermo Fisher, Waltham, MA, USA) and hybrid capture using the xGen Exome Research Panel v1.0 (Integrated DNA Technologies, Inc., Coralville, IA, USA) on the Illumina HiSeq 2500 platform. The generated sequencing reads were 125 bp in length, providing an average coverage depth of approximately 386× across all samples. The raw image files were processed using the base-calling software Illumina 1.7 with default parameters.

### 4.3. Variant Identification and Annotation

After examining the raw data with FastQC v0.18.1, high-quality paired-end reads from each sample were aligned to the GRCh37/hg19 reference genome using the Burrows–Wheeler Aligner (BWA) package. Base quality score recalibration, along with the identification of single nucleotide variants (SNVs) and short insertions or deletions (InDels), was performed using the Genome Analysis Toolkit (GATK 3.8) package and VarScan (version 2.4.0) on improved BAM (.bam) files. Utilizing SAMtools 1.6, high-quality and reliable mutations were obtained after filtering and screening.

Sequence variants, including SNVs and short InDels, were annotated using ANNOVAR software and classified based on their impact on protein function as synonymous, missense, nonsense, stopgain, splice-site mutations, and other genomic features. For coding or splice-site mutations, conservation of the variant site and its predicted effect on protein function were assessed using in-silico tools like SIFT, PolyPhen-2, MutationTaster, and CADD. The sequenced reads were collected, underwent quality filtering, and were aligned to the human reference sequence (UCSC Genome Browser hg196) using the Burrows–Wheeler Aligner. Genotype calling was performed with GATK and VarScan.

### 4.4. Identification of Novel Variant

Systematic family segregation analysis aimed to identify de novo variants, adhering to criteria including (1) the inclusion of protein-altering variants such as frameshift, InDels, missense, stopgain, and intron–exon boundary mutations; (2) the exclusion of synonymous and intron region mutations; and (3) the use of databases like the 1000 Genomes Project, the HapMap CHB (Han Chinese in Beijing, China) population, the National Heart, Lung, and Blood Institute Exome Sequencing Project (ESP), Kaviar_AF database, and the Exome Aggregation Consortium (ExAC) Browser for minor allele frequency (MAF) assessment. Common variants exhibiting a frequency of 0.001 or higher in any of the aforementioned databases were excluded.

### 4.5. Sanger Sequencing

To validate the identified mutations, polymerase chain reaction (PCR) amplification and Sanger sequencing were conducted using standard methods. The relevant sequences from both probands and their parents were amplified, and the resulting fragments were purified using the Agencourt AMPure XP kit (Beckman Coulter, Brea, CA, USA). Sanger sequencing was conducted using the ABI3730xl DNA Sequencer (Applied Biosystems/Thermo Fisher Scientific, Waltham, MA, USA), and the results were analyzed with the Sequencing Analysis 5.2 software (Applied Biosystems/Thermo Fisher Scientific, Waltham, MA, USA). The strength of ectopic splicing sites was assessed with the Human Splicing Finder program. Additionally, the conservation of amino acids was analyzed using the ConSeq server, and structural variations were predicted with the HOPE server.

### 4.6. Molecular Analysis

For determining the pathogenic classification of all variants, a cross-referencing process was conducted with the Human Gene Mutation Database (HGMD), taking into account previous studies. The PSIPRED (v3.3) online software was used to analyze secondary structural variations in candidate genes potentially associated with pathogenic mutations. Furthermore, SWISS-MODEL was used to predict the tertiary structures of the encoded proteins. The Research Collaboratory for Structural Bioinformatics Protein Data Bank (RCSB PDB) and PyMOL software were employed to search for 3D structures and to reconstruct the wild-type and mutant proteins, respectively. The STRING website software (version 12.0) was used to predict protein-to-protein interactions in known skull development pathways, including TGF-β, WNT, BMP, FGF, RA, and Endothelin-1 [31]. The identified mutations were classified according to the HGVS nomenclature guidelines.

### 4.7. Zebrafish Husbandry and Breeding

This study adhered to both local institutional laws and the Chinese law for animal protection. Adult zebrafish were maintained at 28.5 °C, with a 14 h light and 10 h dark cycle, and embryos were staged using standard methods [32]. The Tuebingen and transgenic fish lines, Tg (*sox10*: EGFP) (ID: CZ156, ba2Tg/+), were utilized for the experiments. To prevent pigment formation, zebrafish embryos were treated with 0.2 mM 1-phenyl-2-thio-urea (PTU) 24 h post-fertilization (hpf).

### 4.8. Zebrafish Target Gene Knockout

The CRISPR/Cas9 system was used for knocking out the target gene in zebrafish, adhering to a previously established rapid method for directed gene knockout in F0 zebrafish [33]. Prior to constructing the Genome-Scale Guide Set, guide RNAs (gRNAs) were designed using the CRISPR Design Website (http://chopchop.cbu.uib.no/ (accessed on 1 July 2021)). The gRNA production used the pMD19-gata5_gRNA scaffold as a template, involving the annealing and elongation of a forward primer incorporating a T7 promoter, the guide sequence, the standard chimeric gRNA scaffold (5′-GTT TTA GAG CTA GAA ATA GC-3′), and the standard reverse primer tracr rev (5′-AAA AAA AGC ACC GAC TCG GTG CCA C-3′). The used gRNA sequences are listed in Table S1. Four gRNAs were co-injected alongside Cas9 protein into one-cell stage embryos. The efficacy of the gRNA was confirmed by extracting crude genomic DNA from both the Tuebingen zebrafish embryos (used as the control group) and the zebrafish embryos with a complete target gene knockout. Subsequently, PCR amplification and Sanger sequencing were performed.

### 4.9. Whole-Mount In Situ Hybridization

Whole-mount ISH was performed on zebrafish following established procedures [4]. A template for the *cdc27* antisense probe was prepared by amplifying the full-length zebrafish *cdc27* gene with the forward primer 5′-AGG CTC TAT GCT GAG GTC CA-3′ and the reverse primer 5′-TAA TAC GAC TCA CTA TAG GGA CTG ACA GCC TTC TTT GGG G-3′, including the T7 polymerase sequence. The antisense probe was synthesized with T7 polymerase (Promega, Madison, WI, USA) and a digoxin-NTP mixture (Dig-NTP mix, Roche, Basel, Switzerland). Additionally, probes targeting *crestin*, *dlx2a*, *tbx1*, *barx1*, *sox9a*, and *col2a1a* were used in the whole-mount ISH, with the primer lists provided in Table S2.

### 4.10. Cartilage Staining

Zebrafish embryo cartilages were visualized with Alcian blue staining to highlight the cartilage structure and wheat germ agglutinin (WGA) staining for chondrocyte membranes at 4 days post-fertilization (dpf). Embryos treated with PTU were fixed overnight in 4% paraformaldehyde (PFA) at 4 °C. In the Alcian blue staining procedure, embryos underwent eight one-hour washes in 0.1% Tween-20 H_2_O. After overnight staining of cartilage using 0.015% Alcian blue (SigmaAldrich, Shanghai, China) at 4 °C, embryos were rehydrated with a graded series of alcohols, transitioning to distilled water, and then treated with 0.25% trypsin at room temperature until the tissues became transparent [34]. For WGA chondrocyte membrane staining, embryos were incubated overnight at 4 °C with Alexa Fluor 594-conjugated WGA (Vector, Beijing, China) diluted 1:200, followed by a wash with PBST.

### 4.11. Immunofluorescence

Terminal deoxynucleotidyl transferase dUTP nick-end labeling (TUNEL) assays were conducted to assess cell apoptosis, utilizing the In Situ Cell Death Detection Kit, TMR red (Roche, Shanghai, China), as per the manufacturer’s instructions. Anti-PH3 staining was used to denote cell proliferation. Tg (*sox10*:EGFP) zebrafish embryos were fixed in 4% paraformaldehyde in phosphate buffer and then washed with PBST for 20 min. Immunostaining was performed with PH3 antibodies (1/400; sc-374669, Santa Cruz Biotechnology, Shanghai, China). In both analyses, only cells doubly positive for anti-PH3/TUNEL and EGFP were manually counted, confirming their identity as NCCs. All immunofluorescence images were acquired with a Carl Zeiss LSM710 Confocal Microscope, using consistent settings across all experiments.

### 4.12. Sample Collection for RNA-Sequencing

Zebrafish embryos at 2.5 dpf, exhibiting both mutant and normal phenotypes, were euthanized for total RNA extraction using TRIzol Reagent (Invitrogen, Waltham, MA, USA). Each group consisted of three independent samples, each containing ten embryos. The procedures for assessing sample quality, preparing the RNA library, and conducting RNA-seq were performed as per previously described methods [35]. Subsequently, six sequencing libraries were constructed and underwent sequencing analysis.

### 4.13. Bioinformatics Analysis of RNA-Sequencing

Bioinformatics analysis for RNA-sequencing was conducted using established methods. Initially, raw data were subjected to quality filtering with Trimmomatic (version 0.38), eliminating adapters and low-quality reads and yielding high-quality clean reads. These clean reads were aligned to the reference genome (Danio rerio GRCz11) from the NCBI assembly database using HISAT2 (version 2.1.0), resulting in BAM files for alignment [36]. Subsequently, read counts were obtained using featureCounts, a read summarization program [37]. To ensure robustness, genes with low abundance (summed reads < 10) were filtered out before performing differential expression analysis. The DESeq2 package from Bioconductor was used for gene differential expression analysis [38]. Genes with a fold change ≥ 1.5 and a *p*-value < 0.05 were identified as differentially expressed genes (DEGs).

To analyze DEG enrichment, the KOBAS-i tool was used, focusing on the Kyoto Encyclopedia of Genes and Genomes (KEGG) and Gene Ontology (GO) databases [39]. KEGG enrichment results were visualized with dot plots, and GO enrichment results (*p*-value < 0.05) were presented using bar plots, both generated by the ggplot2 package in R-studio. Jaccard coefficients, calculated in RStudio (Version 1.4.1717), measured the similarity between two KEGG signaling pathways based on shared genes from the enrichment analysis. The resulting network diagram was constructed with Cytoscape software (version 3.8.2). For analyzing hub signaling pathways and genes, the cytoHubba plug-in in Cytoscape was used, employing the MCC (maximal clique centrality) method. The output was exported for visualization [19]. Additionally, GO terms were clustered and pruned using the REVIGO tool, utilizing the *p*-value from KOBASi analysis [40].

### 4.14. Quantitative Real-Time PCR Validation

To validate the RNA-seq results, quantitative real-time PCR (qPCR) was performed using established procedures. Primers were designed with the Primer Premier 6.0 software (PREMIER Biosoft International, San Francisco, CA, USA), and details of sequence IDs, gene names, and amplicon lengths are provided in Table S3. qRT-PCR data analysis followed the protocol by Hellemans et al. [41].

### 4.15. Statistical Analysis

All experiments were performed in triplicate, and unpaired t-tests were employed for data analysis. Results were deemed statistically significant at *p* < 0.05. Consistently, over 85% of the observed embryos exhibited congruent results in all phenotypic observational experiments, and selected images are representative of these findings.

## Figures and Tables

**Table 1:** De novo variants based on VarScan and GATK after filtering.

Family	Variant Number	Gene Number	Gene Names
Family I	2	2	*CDC27*, *SNRNP35*
Family II	1	1	*TTC39C*
Family III	3	2	*ECT2L*, *CDC27*
Family IV	2	2	*ABCA2*, *CTDSP2*
Family V	2	2	*GPM6A*, *COL19A1*
Family VI	2	2	*RBL1*, *GPM6A*
Family VII	1	1	*CDC27*
Family VIII	1	1	*CTBP2*
Family IX	2	2	*COPS7A*, *SNRNP35*
Family X	2	2	*CTBP2*, *CTDSP2*
Family XI	2	2	*COPS7A*, *RBL1*
Family XII	1	1	*CTBP2*

**Table 2:** Annotation of *CDC27* in trio-WES de novo analysis.

Family	Site	Ref	Alt	Function	Exonic Function	AAChange
I	chr17_45219364	A	C	exonic	nonsynonymous	M→R
III	chr17_45258951	A	G	exonic	nonsynonymous	L→P
III	chr17_45258954	A	G	exonic	nonsynonymous	F→S
VII	chr17_45232102	A	C	exonic	stopgain	L→*

Abbreviation: Ref, base of the reference genome; Alt, base of samples; AAChange, changes of amino acid; *, termination codon.

## Data Availability

The data presented in this study are available in the article and the Supplementary Materials.

## Supplementary Materials

The following supporting information can be downloaded at: https://www.mdpi.com/article/10.3390/ijms25094707/s1.

## Abbreviations

Anaphase-promoting complex, cyclosome, subunit 3, ANAPC3; Anaphase-Promoting Complex/Cyclosome, APC/C; cranial neural crest cell, CNCC; differentially expressed genes, DEGs; days post-fertilization, dpf; Exome Aggregation Consortium, ExAC; Exome Sequencing Project, ESP; fibroblast growth factor, FGF; Genome Analysis Toolkit, GATK; Gene Ontology, GO; guide RNA, gRNA; hemifacial microsomia, HFM; Human Gene Mutation Database, HGMD; hours post-fertilization, hpf; in situ hybridization, ISH; insertions or deletions, InDels; Kyoto Encyclopedia of Genes and Genomes, KEGG; neural crest cell, NCC; minor allele frequency, MAF; oculo-auriculo-vertebral spectrum, OAVS; phosphohistone H3, PH3; principal component analysis, PCA; retinoic acid, RA; single nucleotide variants, SNVs; Tetratricopeptide, TPR; transforming growth factor beta, TGF-β; TGF-beta-activated kinase 1, Tak1; Treacher Collins syndrome, TCS; wheat germ agglutinin, WGA; terminal deoxynucleotidyl transferase dUTP nick-end labeling, TUNEL; whole-exome sequencing, WES.
