# Supplementary material for: Functional and Genetic Analyses Unveil the Implication of CDC27 in Hemifacial Microsomia
Source: Int J Mol Sci. 2024 Apr 26;25(9):4707. doi: 10.3390/ijms25094707 (PMC11083823; doi:10.3390/ijms25094707)
Supplement: Supplementary file 1 [file ijms-25-04707-s001.zip › Table S1-The gRNAs and related primer sequences.pdf]

Table S1. The gRNAs and related primer sequences

**Primers**	**Sequence（5'→3'）**
T7-*cdc27* (Exon-1)-sfd	TAATACGACTCACTATAGGAGCCTGGCTACGGAATGA GTTTTAGAGCTAGAAATAGC
T7-*cdc27* (Exon-7)-sfd	TAATACGACTCACTATAgACTGTGACCATAACTCTGC GTTTTAGAGCTAGAAATAGC
T7-*cdc27* (Exon-13)-sfd	TAATACGACTCACTATAgTCGATAGCTCTCTATACGT GTTTTAGAGCTAGAAATAGC
T7-*cdc27* (Exon-18)-sfd	TAATACGACTCACTATAGGATAGTCATCAGGAGTGAC GTTTTAGAGCTAGAAATAGC
T7- *cdkn1a* (Exon-2-1)-sfd	TAATACGACTCACTATAGCCCATTACCGAGTGAACGT GTTTTAGAGCTAGAAATAGC
T7- *cdkn1a* (Exon-2-2)-sfd	TAATACGACTCACTATAGGGCGGAGACTTCCACTGGGAGTTTTAGAGCTAGAAATAGC
T7- *cdkn1a* (Exon-2-3)-sfd	TAATACGACTCACTATAGGGGGTTTCTCCACTTCATGGTTTTAGAGCTAGAAATAGC
T7- *cdkn1a* (Exon-3)-sfd	TAATACGACTCACTATAGgGCTACTGGCCGGATTTGCGGTTTTAGAGCTAGAAATAGC
T7- *tp53* (Exon-2)-sfd	TAATACGACTCACTATAGGCTCGAACCACGGAGCCCTGGTTTTAGAGCTAGAAATAGC
tracr rev	AAAAAAAGCACCGACTCGGTGCCAC
cdc27 (Exon-1)-Forward	CTATTTGAATCGGTTGTGTTCG
cdc27 (Exon-1)-Reverse	GTCTTCTACCGTAACTGCGACC
cdc27 (Exon-7)- Forward	GGTGTAGTTTTGGAATTTTGCC
cdc27 (Exon-7)- Reverse	ATAACCGCAGATTCTGTCTTGG
cdc27 (Exon-13)- Forward	GGTGAAATCAATTACCAAAGGC
cdc27 (Exon-13)- Reverse	TTTTGTCCATGTCAGTGAGGTC
cdc27 (Exon-18)- Forward	CTCCTGAATCAAATGTTCGTCA
cdc27 (Exon-18)- Reverse	CAACAAACAGCATTACACCGAG
cdkn1a (Exon-2-1,2)- Forward	ACCCCTAAAACTGTCATCGCTA
cdkn1a (Exon-2-1,2)- Reverse	ACCTGTGATGTTGGTCTGTTTG
cdkn1a (Exon-2-3)- Forward	TTGCGTCTGAGAAACCACTAGA
cdkn1a (Exon-2-3)- Reverse	TGAAGGTAGATGCAGGTCAAGA
cdkn1a (Exon-3)- Forward	ACAGACCAACATCACAGGTCAG
cdkn1a (Exon-3)- Reverse	AGGAATGGATCTTTCCAGAACA
tp53 (Exon-2)- Forward	GGCCATTGTCATTTTTCGTATT
tp53 (Exon-2)- Reverse	AATTACACACACTTGCCTCCCT

Note: The black underline indicates the T7 promoter sequence; The blue sequence indicates the main sequence of the target (excluding PAM); The red sequence indicates the fixed sequence of gRNA scaffold; The "g" refers to the adjustment for the first base of the main sequence is not "g".
