# Supplementary material for: Functional and Genetic Analyses Unveil the Implication of CDC27 in Hemifacial Microsomia
Source: Int J Mol Sci. 2024 Apr 26;25(9):4707. doi: 10.3390/ijms25094707 (PMC11083823; doi:10.3390/ijms25094707)
Supplement: Supplementary file 1 [file ijms-25-04707-s001.zip › Table S2-Probe primers of in situ hybridization.pdf]

Table S2. Probe primers of *in situ* hybridization

Primers	Sequence（5'→3'）
cdc27-F	GAGCCCTGGTGTGTAGTTGG
cdc27-R	GATTCCTCCACCTCCGTTGA
cdc27-T7 R	TAATACGACTCACTATAGGGGATTCCTCCACCTCCGTTGA
crestin-F	GAGAAGCCCTCATCAGAGAGTTTG
crestin-R	GTTGCTTGTCAGGCAGAATCAGG
crestin-T7 R	TAATACGACTCACTATAGGGGTTGCTTGTCAGGCAGAATCAGG
col2a1a-F	TCTGAAGTCCATCAACGGGC
col2a1a-R	TTTTCCGTCACGCTAAACGC
col2a1a-T7 R	TAATACGACTCACTATAGGGTTTTCCGTCACGCTAAACGC
sox9a-F	CCTCGACCCCTACCTGAAGA
sox9a-R	GGCGGGAGGTATTGGTCAAA
sox9a-T7 R	TAATACGACTCACTATAGGGGGCGGGAGGTATTGGTCAAA
barx1-F	CTGGGCGGATCAGACTTCTC
barx1-R	GCTTCTCGTGTCCTCTCCTG
barx1-T7 R	TAATACGACTCACTATAGGGGCTTCTCGTGTCCTCTCCTG
dlx2a-F	CACAGTTCTGCTTTGCGTCG
dlx2a-R	CCCAAGTCGGCAGAGTCAAA
dlx2a-T7 R	TAATACGACTCACTATAGGGCCCAAGTCGGCAGAGTCAAA
foxd3-F	CCTACTCGTACATCGCCCTC
foxd3-R	CCGGGTTAAGGACAGGGAC
foxd3-T7 R	TAATACGACTCACTATAGGGCCGGGTTAAGGACAGGGAC
nkx2.3-F	TCGTGTTTTCTCGGAGGTGG
nkx2.3-R	GCGCATTAGTGGACGTGTTC
nkx2.3-T7-R	TAATACGACTCACTATAGGGGCGCATTAGTGGACGTGTTC
fgf3-F	GAATCTCTGGCTCCGAGGCT
fgf3-R	GCCGCTGACTCTCTCTAAGC
fgf3-T7-R	TAATACGACTCACTATAGGGGCCGCTGACTCTCTCTAAGC
tbx1-F	GCAGCTGTCCCATTTTTGCG
tbx1-R	ACGGCGGTAAATCTGGTCTC
tbx1-T7-R	TAATACGACTCACTATAGGGACGGCGGTAAATCTGGTCTC
