# Supplementary material for: Functional and Genetic Analyses Unveil the Implication of CDC27 in Hemifacial Microsomia
Source: Int J Mol Sci. 2024 Apr 26;25(9):4707. doi: 10.3390/ijms25094707 (PMC11083823; doi:10.3390/ijms25094707)
Supplement: Supplementary file 1 [file ijms-25-04707-s001.zip › Table S3-Primers of qRT-PCR.pdf]

Table S3. Primers of qRT-PCR

**Primers**	**Sequence（5’→3’）**
gapdh-F	GGGTGATGCAGGTGCTACTT
gapdh-R	AAAGGAGCCAGGCAGTTGG
cdc27-F	AGGCTCTATGCTGAGGTCCA
cdc27-R	TCCTCCGGTCAGGATCTGTT
tp53-F	GTGGCTCTTGCTGGGACATC
tp53-R	CAGGTCCGGTGAATAAGTGCAA
cdkn1a-F	GAGCTTCGACTTTGCGTCTG
cdkn1a-R	CTTGGCTTGGTAGAAATCTGTG
gadd45aa-F	GACCCTGATAACGTGGTGCT
gadd45aa-R	GCATGTGGAACCGTGACCA
ccng1-F	CGATACACTGCAAGCTGAAACTG
ccng1-R	GCTGGAAGAGGTCGTGATTGA
serpine1-F	GGTGCATTGCTCTTTAGCGG
serpine1-R	GGCAGTCCAAGCTATGCTGA
sox9a-F	TTTGACCAATACCTCCCGCC
sox9a-R	AGGAACCGTAGCTGATGTGC
col2a1a-F	TGACCAGGAAGAGTTTGGCG
col2a1a -R	TTTGCACCAAGTGACCCGAT
