# Supplementary figures and images for: Functional and Genetic Analyses Unveil the Implication of CDC27 in Hemifacial Microsomia
Source: Int J Mol Sci. 2024 Apr 26;25(9):4707. doi: 10.3390/ijms25094707 (PMC11083823; doi:10.3390/ijms25094707)

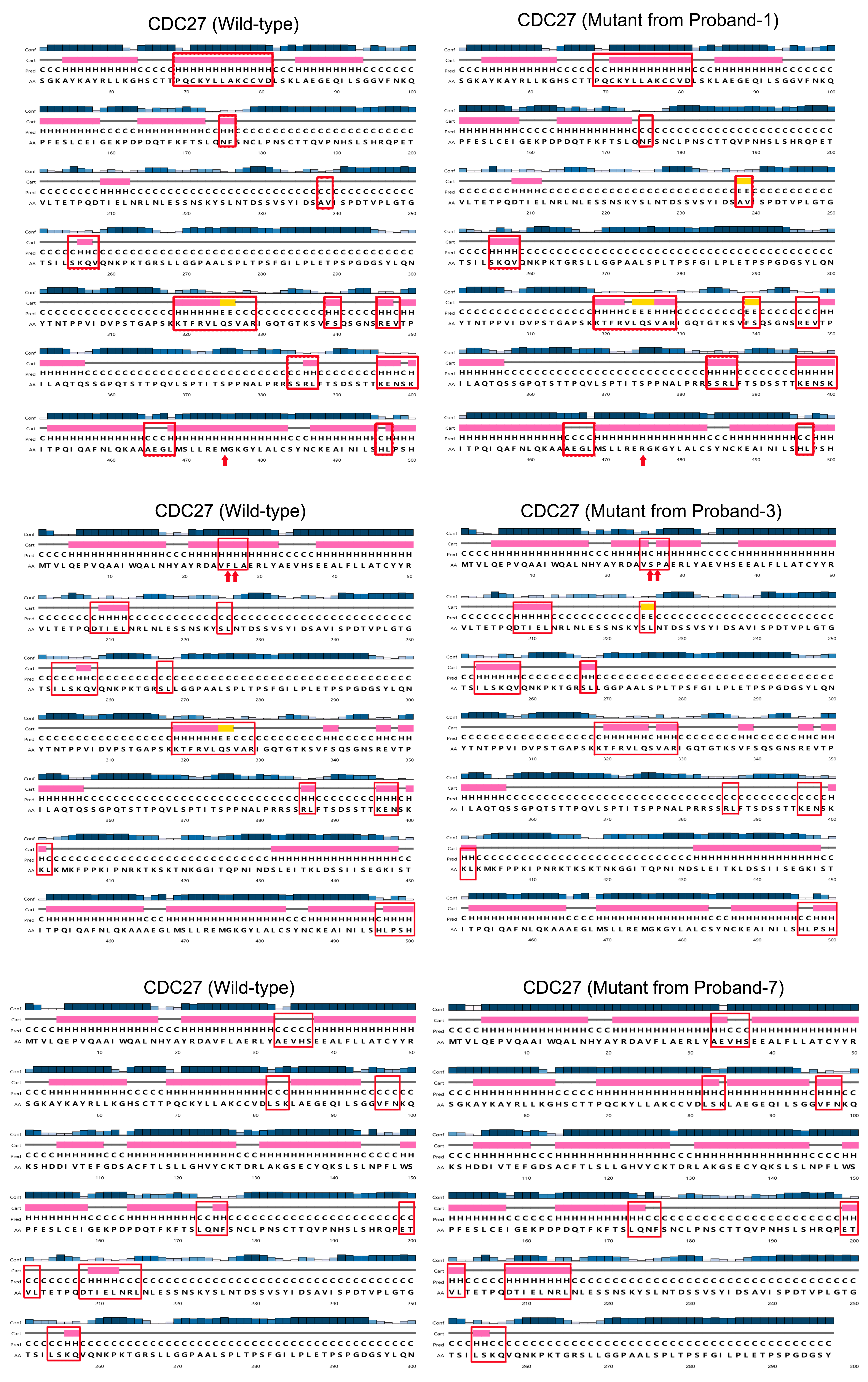

Supplement: Supplementary file 1 [file ijms-25-04707-s001.zip › figure S1.tif]

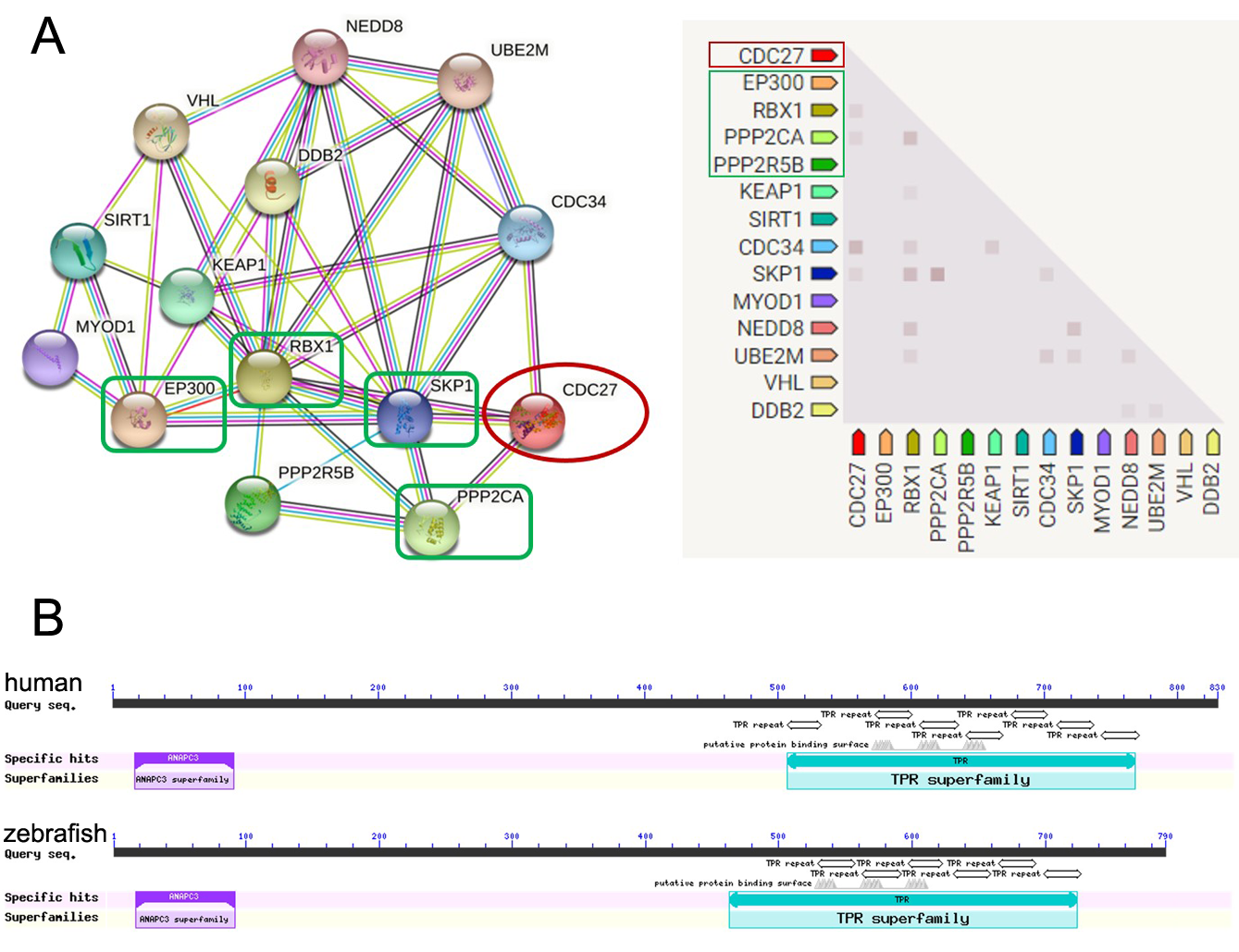

Supplement: Supplementary file 1 [file ijms-25-04707-s001.zip › figure S2.tif]

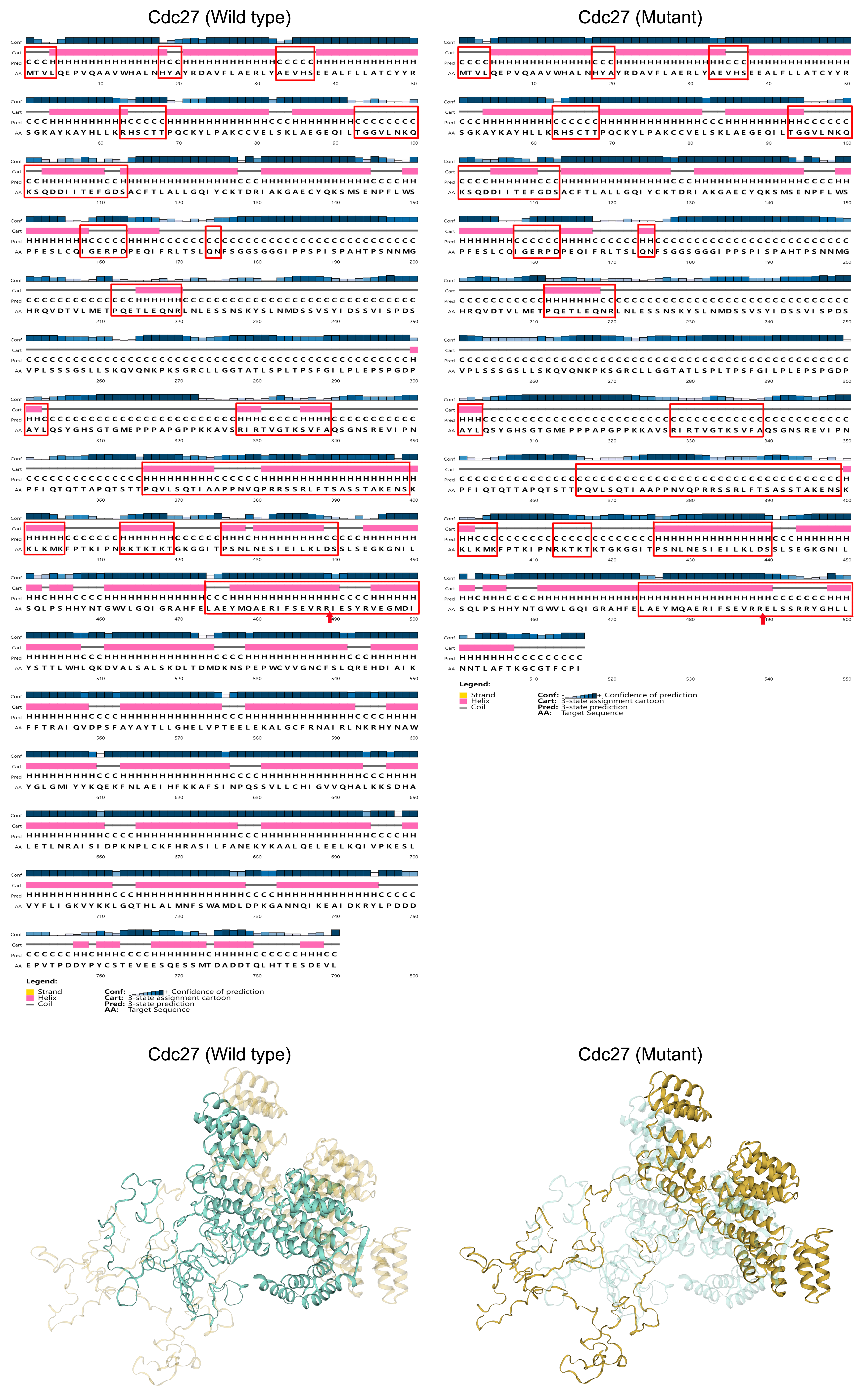

Supplement: Supplementary file 1 [file ijms-25-04707-s001.zip › figure S3.tif]

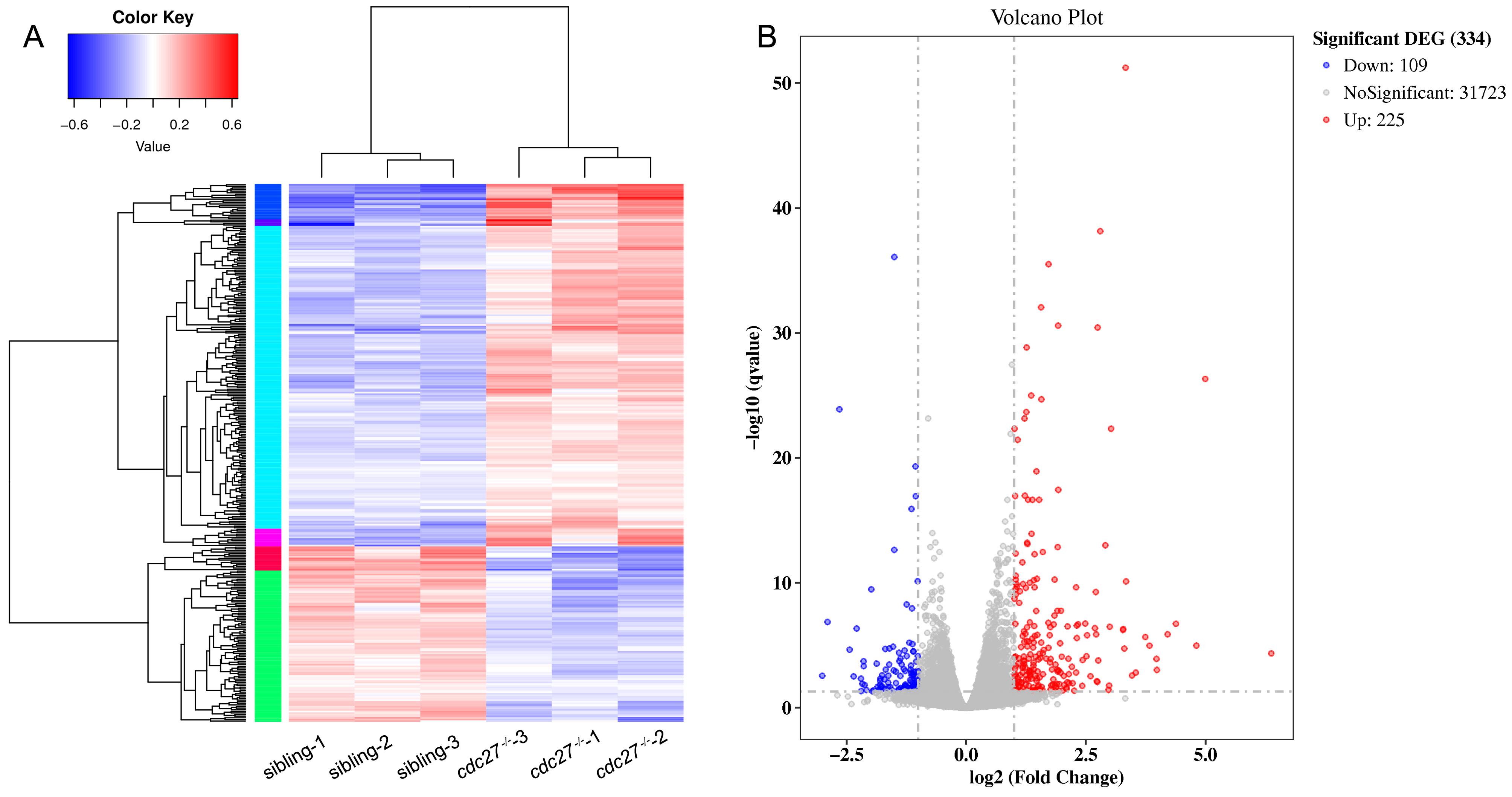

Supplement: Supplementary file 1 [file ijms-25-04707-s001.zip › figure S4.tif]

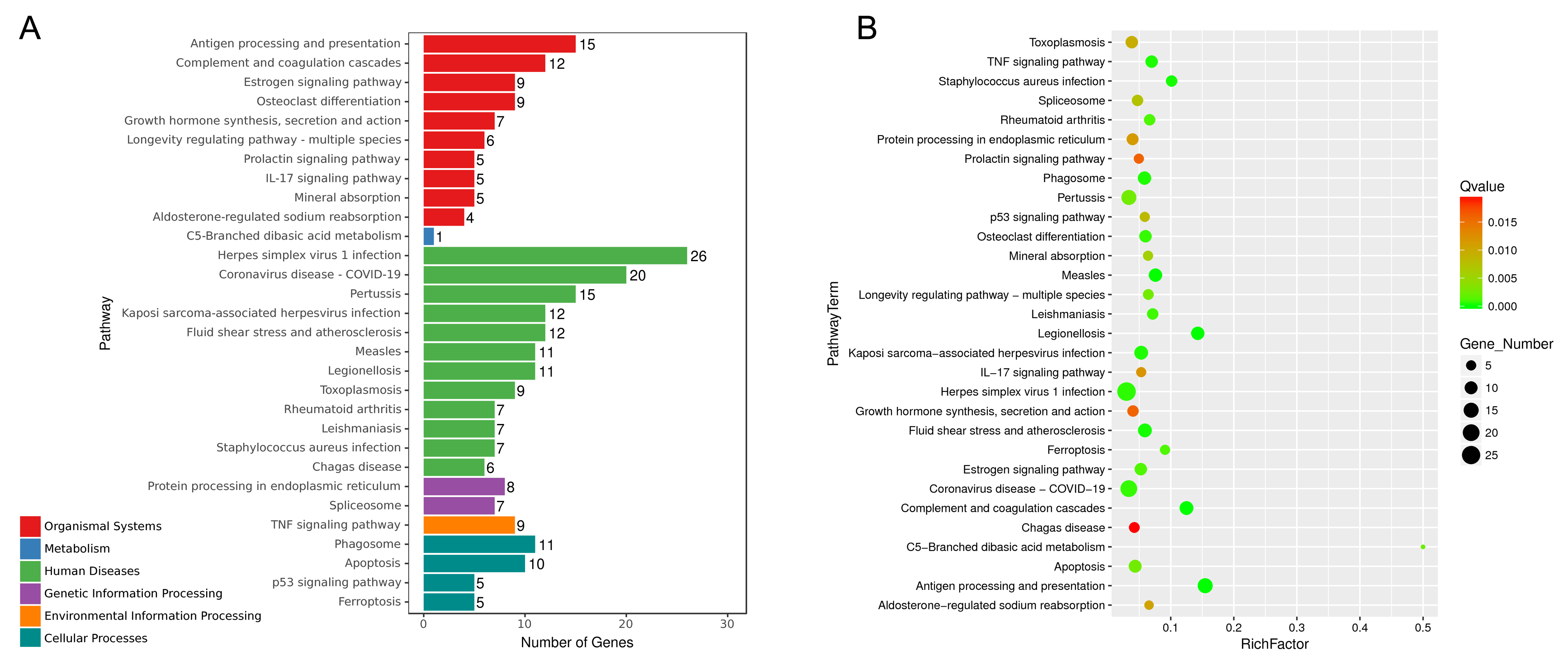

Supplement: Supplementary file 1 [file ijms-25-04707-s001.zip › figure S5.tif]

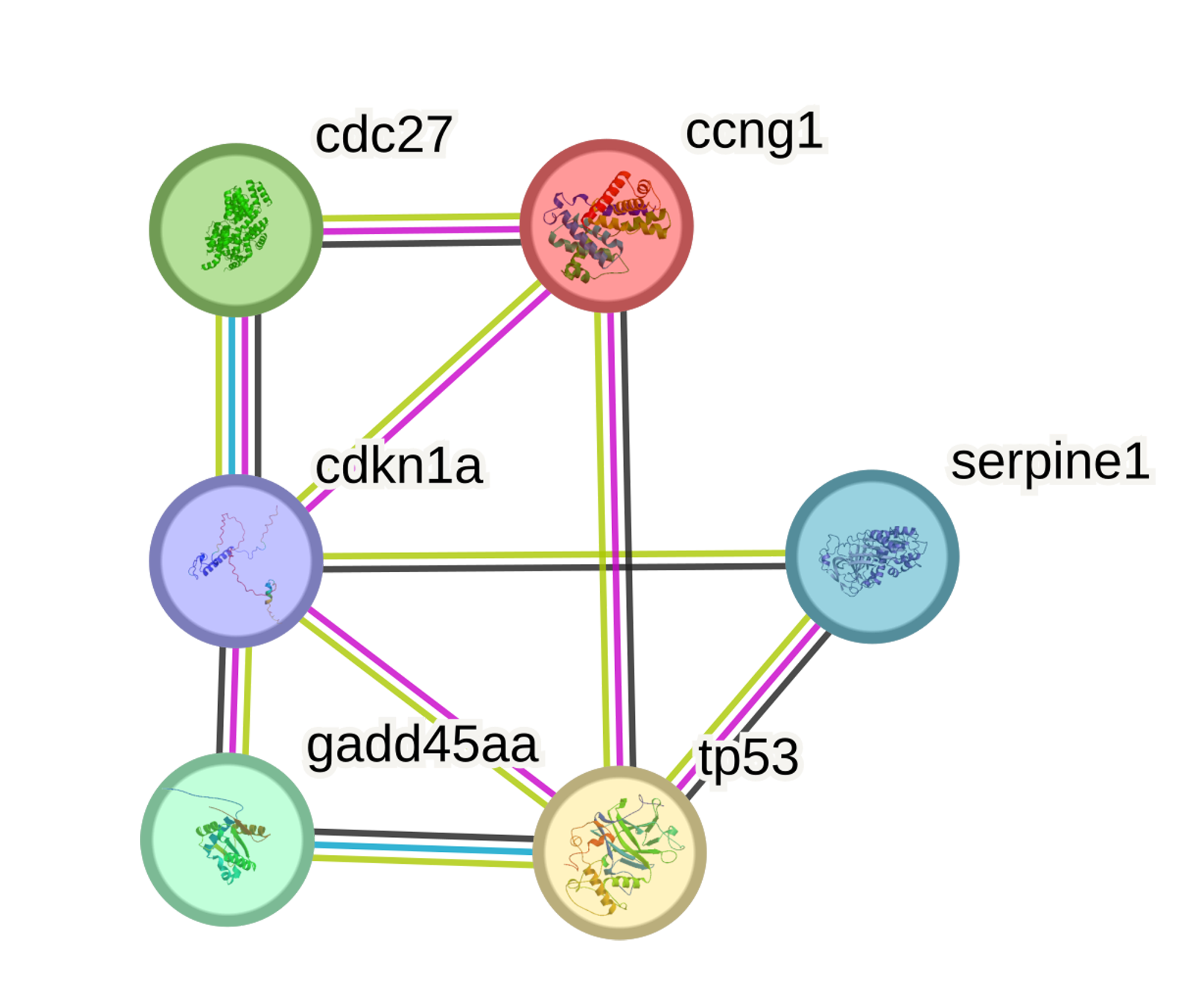

Supplement: Supplementary file 1 [file ijms-25-04707-s001.zip › figure S6.tif]
